# Supplementary material for: Stacking Model‐Based Classifiers for Dealing With Multiple Sets of Noisy Labels
Source: Biom J. 2025 Mar 12;67(2):e70042. doi: 10.1002/bimj.70042 (PMC11898607; doi:10.1002/bimj.70042)
Supplement: Supplementary file 1 — Supporting Information [file BIMJ-67-e70042-s001.zip › density-based-ensemble-model-main/competitors_real_data/netlab-master/netlab-master/help/pca.htm]

Netlab Reference Manual pca


# pca

## Purpose

Principal Components Analysis

## Synopsis

```
PCcoeff = pca(data)
PCcoeff = pca(data, N)
[PCcoeff, PCvec] = pca(data)
```

## Description

`PCcoeff = pca(data)` computes the eigenvalues of the covariance
matrix of the dataset `data` and returns them as `PCcoeff`. These
coefficients give the variance of `data` along the corresponding
principal components.

`PCcoeff = pca(data, N)` returns the largest `N` eigenvalues.

`[PCcoeff, PCvec] = pca(data)` returns the principal components as
well as the coefficients. This is considerably more computationally
demanding than just computing the eigenvalues.

## See Also

`eigdec`, `gtminit`, `ppca`

---

**Pages:**
Index


---

Copyright (c) Ian T Nabney (1996-9)
